# Water-drinking Test and Pharmacologic Mydriasis as Provocative Tests in Primary Angle Closure Suspects

**DOI:** 10.18502/jovr.v14i3.4782

**Published:** 2019-07-18

**Authors:** Reza Razeghinejad, M. Hossein Nowroozzadeh

**Affiliations:** ^1^Glaucoma Service, Wills Eye Hospital, Philadelphia, PA, USA; ^2^Poostchi Ophthalmology Research Center, Shiraz University of Medical Sciences, Shiraz, Iran

**Keywords:** Intraocular Pressure, Pharmacologic Mydriasis, Primary Angle Closure Suspect, Water-drinking Test

## Abstract

**Purpose:**

To compare the water-drinking test (WDT) and pharmacologic mydriasis as provocative tests in patients with primary angle closure suspect (PACS).

**Methods:**

This observational non-randomized comparative study evaluated changes in intraocular pressure (IOP) in 21 patients with PACS who underwent pharmacologic mydriasis and compared it with IOP changes in 26 patients given the WDT. Ocular biometric and anterior chamber parameters were also assessed. Tests were repeated on the same patient two weeks after performing laser peripheral iridotomy (LPI).

**Results:**

The mean age ± standard deviation was 60 ± 7 and 57 ± 9 years in the mydriasis and WDT groups, respectively (*P* = 0.201). Before LPI, both provocative tests were associated with a significant increase in IOP (mydriasis: 15.1 ± 3.1 to 16.6 ± 3.5 mmHg, *P* = 0.025; WDT: 16.2 ± 2.8 to 18.5 ± 3.3 mmHg, *P* < 0.001). However, the IOP changes were not statistically different between groups (*P* = 0.102). After LPI, only the WDT group showed a continued significant IOP elevation after the test (mydriasis: 16.4 ± 3.3 to 16.7 ± 3.5 mmHg, *P* = 0.569; WDT: 14.9 ± 3.0 to 17.8 ± 4.1 mmHg, *P* < 0.001). The post-test IOP change was significantly greater in the WDT than in the mydriasis group (3.0 versus 0.3 mmHg, respectively; *P* = 0.002). Step-wise multiple regression analysis verified the type of provocative test as the only independent factor affecting the post-test IOP change after LPI (regression coefficient: 2.664; *P* = 0.002).

**Conclusion:**

Pharmacologic mydriasis and the WDT had similar IOP elevation before LPI, but after LPI, IOP elevation was much greater in the WDT group.

##  INTRODUCTION

Primary angle closure glaucoma (PACG) is believed to be one of the leading causes of irreversible blindness with approximately 20 million people experiencing PACG worldwide.^[1,2,3]^ The spectrum of angle closure disease includes a narrow angle with 180° or more of iridotrabecular apposition (primary angle closure suspect [PACS]), a narrow angle with increased intraocular pressure (IOP) or peripheral anterior synechiae (primary angle closure, [PAC]), and PAC with optic neuropathy or PACG.^[4]^ It has been reported that approximately 10% of PACS patients eventually develop glaucoma.^[2]^ While there is some evidence that a prophylactic laser peripheral iridotomy (LPI) significantly reduces the risk of angle closure glaucoma in the contralateral eye of an individual with angle closure glaucoma, there is much confusion regarding the need for a prophylactic LPI for patients with asymptomatic narrow angles detected through gonioscopy. Although LPI is an effective preventive therapy, we must consider the large number of candidates involved and the burden and cost of treating ten-folds for each eye that receives an actual benefit. Therefore, identifying PACS eyes that would benefit from LPI through appropriate imaging or provocative tests is desirable. Various provocative tests including the dark-room provocative test and pharmacologic mydriasis have been used to identify narrow angle eyes at risk of angle closure.^[5]^ Pharmacologic or dark-induced mydriasis leads to a relative pupillary block and increased iridotrabecular contact. The prone position in the dark room test may induce forward movement of the lens and enhance the effect of the relative pupillary block.^[6]^ However, this provocative test has a low sensitivity and positive predictive value in detecting eyes susceptible to angle closure glaucoma.^[7]^ Therefore, we need to identify more appropriate provocative tests to determine PACS patients with the greatest risk of PACG and learn more about the mechanism of angle closure formation.

The water-drinking test (WDT) is used as a provocative test for assessing the outflow facility of aqueous humor in patients with primary open-angle glaucoma. Choroidal expansion is a suggested mechanism of IOP elevation in WDT.^[8,9]^ Choroidal expansion has also been suggested as one of the mechanisms involved in PACG development.^[10]^ The expansion of the choroid after WDT could push the iris-lens diaphragm forward and result in a decrease in anterior chamber volume.^[8,11]^ Therefore, this test has the potential to be a provocative test in PACS. This study aimed to evaluate the changes in the IOP, ocular biometric, and anterior chamber parameters in PACS patients who underwent WDT or pharmacologic mydriasis, before and after LPI.

##  METHODS

This observational non-randomized comparative study enrolled 47 consecutive patients with PACS who were referred to the glaucoma clinic of a tertiary eye care center. The first 21 referred patients received the pharmacologic mydriasis test and the next 26 patients underwent WDT. All procedures performed in this study were in accordance with the 1964 Helsinki Declaration and its later amendments. The study was approved by the local Ethics Committee and informed consent was obtained from all patients. The patients underwent a comprehensive ophthalmologic examination including slit lamp biomicroscopy, Goldmann applanation tonometry (Haag Streit AG, Bern, Switzerland), indentation gonioscopy using a Sussman 4-mirror gonio lens (Volk Optical Inc., Mentor, OH, USA), and stereoscopic assessment of the optic disc with a 90 diopter lens (Volk Optical Inc., Mentor, OH, USA). Subjects were classified as PACS if they presented ≥ 180${}^{o}$ of iridotrabecular contact, without peripheral anterior synechiae in indentation gonioscopy, glaucomatous optic neuropathy, or IOP > 22 mmHg.^[12]^ The exclusion criteria consisted of a history of ocular trauma, prior intraocular or refractive surgery, any intraocular disorder except cataract, secondary angle closure glaucoma, evidence of active keratitis or cornel pathology precluding gonioscopy and fundus examination, and the use of miotics or anticholinergics, pregnancy, hypertension, cardiac or kidney diseases, history of urinary retention, or inability to cooperate with any of the study measurements.

###  Water-drinking Test (WDT)

To perform the WDT, the patients refrained from food and fluid intake for three hours preceding the test. Patients were instructed to drink one liter of bottled water within five minutes. All WDTs were performed between 12 to 2 pm. The original WDT developed for primary open angle glaucoma detection and the majority of recent studies assessing the effect of WDT on IOP fluctuation used one liter of water for this purpose.^[13,14,15,16]^ Therefore, we opted to use one liter instead of the 10–15 mL/kg reported by other studies. The measurements were obtained at baseline and at 30 minutes after drinking the water.^[8]^ The patients then underwent LPI using a commercial ophthalmic Nd: YAG laser system (Nidek YC-1800, Nidek Inc., Japan) one week after the WDT. The laser parameters were as follows: one pulse with a power of 4–6 mJ performed approximately 30 minutes after applying one drop of 2% pilocarpine eye drops.^[17]^ Two weeks after LPI, all measurements were repeated before and after 30 minutes of WDT.

###  Mydriasis Test

The measurements were obtained before and after the induction of pharmacologic mydriasis. In order to eliminate any possible effects of cycloplegic agents on the ciliary muscles and lens position, mydriasis was achieved with phenylephrine 5% drops applied every five minutes for a total of two applications. LPI was performed the week following the mydriasis test. All measurements were repeated before and after mydriasis, two weeks after LPI.

###  Measurements

The main outcome measure was IOP change after the tests. An increase in IOP of ≥ 6 mmHg from baseline was considered a positive result on both provocative tests.^[18]^ Secondary outcomes were refraction ocular biometric parameters obtained using Lenstar LS 900 (Haag-Streit AG, Koeniz, Switzerland) and Pentacam HR (Oculus, Wetzlar, Germany) optical biometers. The following parameters were collected from the Lenstar biometer: mean keratometry (Km), keratometric astigmatism (Ka), central corneal thickness (CCT), axial length (AL), and lens thickness (LT). The Pentacam HR was used to obtain Km, central anterior chamber depth (ACD; from the endothelium to the anterior lens surface), anterior chamber volume, and inferior anterior chamber angle. The following factors were calculated from the Lenstar recordings: lens-axial length factor [LAF = (LT/AL) × 10]; lens position (LP = ACD + 0.5 × LT); and relative lens position [RLP = (LP/AL) × 10]. Each instrument was calibrated at the beginning of the study, and at regular intervals afterward (as per manufacturer's recommendations). All measurements were performed by the same experienced investigator using the criteria provided by the manufacturer of each device.

###  Statistical Analysis

IBM SPSS Statistics software version 21 (SPSS Inc., Chicago, IL) and MedCalc version 12.2.1 (MedCalc Software, Mariakerke, Belgium) were used for statistical analyses. Data from one eye of each patient, chosen at random, were included in the analysis. Descriptive data were presented as mean (± standard deviation). The normality of data was assessed using the Kolmogorov-Smirnov test. The paired-samples *T*-test (or its nonparametric counterpart, the Wilcoxon-signed Rank test) was used to compare data before and after each test. The independent-sample *T*-test (or Mann-Whitney U test) was used to compare post-test changes between different groups. Linear regression analysis was used to evaluate the effects of different parameters on the test results. A *P*-value of less than 0.05 was considered statistically significant.

##  RESULTS

**Figure 1:**
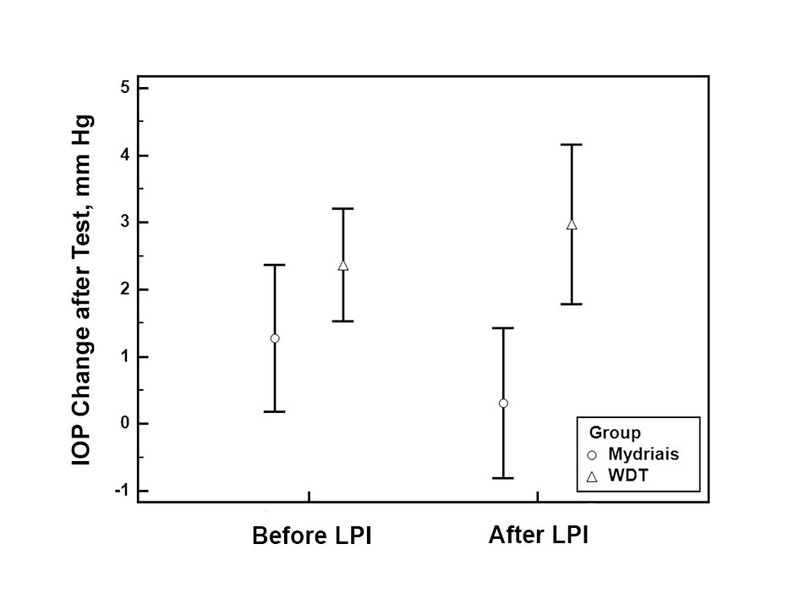
Comparison of intraocular pressure (IOP) changes after the water-drinking test (WDT) and pharmacologic mydriasis in primary angle closure suspect patients before and after laser peripheral iridotomy (LPI).

Data from 21 PACS patients in the pharmacologic mydriasis group were compared with 26 patients in the WDT group. Table 1 summarizes the baseline characteristics of patients in each group. Before LPI, both provocative tests were associated with a significant IOP elevation (Table 2). IOP elevation in the WDT group was almost twice of the mydriasis group (2.4 versus 1.3); however, the difference was not statistically significant. Following LPI, the WDT group had a significant increase in IOP after the test, while the pharmacologic mydriasis group did not [Table 2; Figure 1]. The post-LPI WDT IOP-change was significantly greater in the WDT than the mydriasis group (3.0 versus 0.3 mmHg, respectively; *P* = 0.002). Before LPI, two (7.6%) patients in the WDT and one (4.7%) in the mydriasis group had positive test results (≥ 6 mmHg increase in IOP), but after LPI, seven (26.9%) patients in the WDT group and none of the patients in the mydriasis group had positive test results (P = 0.01; Chi-Square test).

**Table 1:** Baseline characteristics of patients suspected of primary angle closure that underwent provocative testing with either the pharmacologic mydriasis or water-drinking test


**Provocative test**	**Pharmacologic mydriasis**	**Water-drinking test**	**** ***P*** **-value**
Number	21	26	
Age, years	60 ± 7	57 ± 9	0.201
Sex, M/F	4/17	22/4	< 0.001
Spherical equivalent of refraction, D	1.3 ± 1.2	0.6 ± 1.0	0.036
Axial length, mm	22.5 ± 0.6	22.4 ± 0.8	0.731
Anterior chamber depth, mm	2.20 ± 0.21	2.24 ± 0.24	0.579
Anterior chamber volume, μm${}^{3}$	88.2 ± 13.7	94.3 ± 17.6	0.200
Anterior chamber angle, degrees	25.6 ± 7.0	28.2 ± 5.5	0.167
Central corneal thickness, μm	527 ± 35	535 ± 28	0.426
Mean keratometry, D	44.6 ± 1.2	44.7 ± 1.7	0.736
	
	
D, diopter; F, female; M, male; mm, millimeter; μm, micrometer			

**Table 2:** Comparison of pharmacologic mydriasis and water-drinking tests as provocative tests in patients suspected of primary angle closure before and after laser peripheral iridotomy


**IOP (mmHg) ** ***before*** ** laser peripheral iridotomy **				
	**Before test**	**After test**	**** ***P*** **-value**	Changes*
Pharmacologic mydriasis	15.1 ± 3.1	16.6 ± 3.5	0.025	1.3 ± 2.2
Water-drinking Test	16.2 ± 2.8	18.5 ± 3.3	< 0.001	2.4 ± 2.1
*P*-value		0.102
**IOP (mmHg) ** ***after*** ** laser peripheral iridotomy**				
Pharmacologic mydriasis	16.4 ± 3.3	16.7 ± 3.5	0.569	0.3 ± 2.5
Water-drinking test	14.9 ± 3.0	17.8 ± 4.1	< 0.001	3.0 ± 2.9
*P*-value		0.002
	
	
*Defined as: Post-test IOP – Pre-test IOP			
IOP, intraocular pressure			

**Table 3:** Regression analysis of possible factors affecting IOP change after provocative tests of water-drinking test and pharma- cologic mydriasis in patients with primary angle closure suspect after laser peripheral iridotomy


**Factors**	**Simple Linear Regression**	**Multiple Regression**	
	**** ***P*** **-value**	**Coefficient**	**** ***P*** **-value**
Age	0.332	
Sex	0.094*	
Test (WDT vs. pharmacologic mydriasis)	0.002*	2.664	0.002
Baseline IOP	0.225	
SE Refraction	0.359	
Anterior chamber depth	0.406	
Anterior chamber volume	0.301	
Anterior chamber angle	0.278	
Axial Length	0.565	
Central corneal thickness	0.814	
Mean keratometry	0.118	
	
	
*Factors with *P*-value less than 0.1 were entered into a multiple stepwise regression model			
IOP, intraocular pressure; SE, spherical equivalent; WDT, water-drinking test			

**Figure 2:**
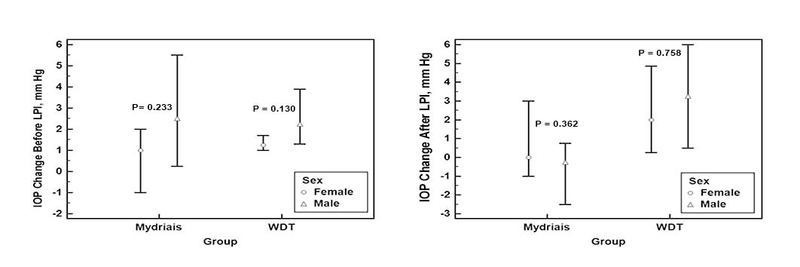
Effect of sex on intraocular pressure (IOP) changes after the water-drinking test (WDT) and pharmacologic mydriasis in primary angle closure suspect patients before and after laser peripheral iridotomy (LPI).

There were no significant differences in biometric parameters for both tests before and after LPI (data not shown). Because the two studied groups were not similar in terms of sex and refraction, we conducted a linear regression analysis to assess the effects of different factors on the post-test IOP changes after LPI (Table 3). In simple regression analysis, sex, and the type of the provocative test appeared to be associated with the observed post-test IOP changes after LPI (*P* < 0.1). Figure 2 shows the effects of sex on IOP changes after provocative tests were performed before and after LPI. Sex had no significant association with IOP changes either before or after LPI (Mann-Whitney U test). In line with this finding, step-wise multiple regression analysis showed that the sex had no independent effect, and the type of the provocative test was the only independent factor affecting the post-test IOP changes after LPI (regression coefficient: 2.664; *P* = 0.002).

##  DISCUSSION

Our results showed that the amount of IOP elevation after pharmacologic mydriasis was less than WDT-IOP elevation before and after LPI. There was no statistically significant difference between the pharmacologic mydriasis and WDT groups in terms of IOP elevation before LPI, but after LPI, the increase in IOP was statistically significantly greater in the WDT group. After LPI, the IOP changes were lower in the mydriasis group compared with before LPI measurements, while the IOP changes were greater in the WDT group in measurements obtained after LPI than before LPI.

After LPI, the IOP changes in the mydriasis group were lower than before LPI (1.3 versus 0.3 mmHg), while the differences were greater in the WDT group (2.4 versus 3.00 mmHg). Moreover, there was a greater number of patients with positive WDT test results after LPI compared to before LPI. The lower post-LPI IOP change in the mydriasis group could be attributed to the resolution of the pupillary block component of IOP elevation. A higher change in WDT-IOP after LPI compared to before LPI has been reported previously in PACS patients.^[19]^ The WDT-IOP elevation mechanisms may not be dependent on the pupillary block, but it is directly associated with the outflow capability of aqueous humor. In a previous study, in 16 out of 18 eyes subjected to argon laser iridotomy, there was evidence of decreased trabecular outflow after LPI in tonography.^[20]^ The decreased outflow may be explained by the possibility of damage from pigment release and inflammation after LPI to the already somehow impaired trabecular meshwork function due to the long-term apposition to the iris.

The WDT is a stress test that was initially developed to screen for open-angle glaucoma; however, its diagnostic capabilities showed that the WDT lacked the sensitivity and specificity needed to be a reliable screening test. Currently, the WDT is used to evaluate the aqueous outflow facility and the efficacy of surgical and medical glaucoma management, in addition to predicting the diurnal IOP peak.^[21,22]^ Sympathetic stimulation via yet-to-be-determined mechanisms has been suggested to be responsible for the WDT-IOP elevation.^[23]^ Increased iris thickness, owing to sympathetic stimulation of the iris dilator muscle and its resulting circumferential folding, may prompt angle closure in patients with narrow angle glaucoma. Moreover, a significant increase in choroidal thickness and IOP elevation and a decrease in anterior chamber depth after WDT have been observed in angle closure but not in open angle eyes.^[8]^ The lack of any changes in the ocular biometric parameters in our patients of both groups is in line with other studies on the angle closure patients.^[24,25,26,27]^ In a group of fellow eyes of 21 patients with acute primary angle closure and in 17 patients with PACG, the ACD did not change following WDT.^[26]^ The study by Poon et al on PACG and primary open-angle glaucoma revealed no association between WDT-IOP changes and anterior chamber depth and axial length.^[25]^ In a study on patients with PACS, PAC, and PACG, the positive dark room and mydriasis tests were defined as an increase in IOP of 6 mmHg. There was no difference in the anterior chamber depth between positive and negative testers (1.95 versus 1.89 mmHg, *P* = 0.3) with the dark room test, but it was shallower in the patients with a positive mydriasis test (1.77 versus 1.96 mmHg, *P* = 0.010). This difference could have resulted from the limited number of patients in each subgroup because the suggested mechanisms underlying both tests are similar.^[27]^ In a study on PACS patients, no changes in ocular biometric or anterior chamber parameters including iridocorneal angle after peripheral iridotomy and/or pharmacologic mydriasis except for increments in anterior chamber volume were observed.^[24]^

The mechanisms involved in WDT-IOP elevation are still unknown and may be different in open and closed angle eyes. The resolution of the pupillary block after LPI and the lower IOP elevation observed following pharmacologic mydriasis and the greater IOP elevation with WDT may exclude the possibility of the pupillary block as the main mechanism of WDT-IOP elevation in patients with closed angle eyes. Long-term follow-up of patients for IOP elevation may clarify the role of WDT in this group of patients.

Although it has been suggested that LPI be performed for narrow angles that fulfill the criteria of PACS, the decision of which subjects require prophylactic LPI remains highly subjective. It has been estimated that approximately one out of ten patients with anatomically narrow angles develop angle closure,^[28]^ therefore, the option of treating all patients with narrow angles would result in a large majority undergoing unnecessary procedures and a waste of resources. There is a great demand to develop a test that could identify PACS likely to progress to PAC. It has been suggested that angle closure may often be caused by other mechanisms, which would not be resolved by an iridotomy, such as peripheral iris crowding, a plateau iris or lens-related angle closure, and other unknown mechanisms.^[29]^ Interestingly, it has been reported that the extent of PAS did not correlate with the rise in IOP seen on provocative tests such as the dark room test, pharmacologic mydriasis, and the Valsalva test in patients with PAC.^[18]^ Parameters identifying eyes that continue to develop angle closure after an iridotomy would lead to better management of PAC. Although LPI is claimed to prevent PACS progression to PAC or PACG, it is not guaranteed, as some patients progress despite successful LPI. IOP elevation after WDT, but not after pharmacologic mydriasis, probably involves mechanisms other than pupillary block.

Limitations of our study include the relatively small sample size; thus our results require validation in future studies involving more patients. In addition, inherent variability of IOP measurements because of diurnal changes and device repeatability issues might affect the results. We limited IOP measurements to the same time frame on each day to minimize the confounding effects. Another limitation is the lack of angle grading information. Because the purpose of this study did not include the evaluation of angles before and after the LPI, we did not include the gonioscopy grading. However, patients in both groups had similar anterior chamber depth, anterior chamber angle width, anterior chamber volume, and axial length, which could indicate a lack of notable differences in angle configuration between the two groups. Performing pharmacologic mydriasis before LPI may be regarded as an ethical limitation of the current study as it may induce an acute angle closure attack. None of the eyes in our study developed an acute attack during the tests and the IOPs prior to and following the LPI were very similar. Diagnostic mydriasis appears to be relatively safe in patients with narrow angle. The risk of acute angle closure attack after pupillary dilation has been reported to be 0.03–0.3%.^[30,31]^ Moreover, in a study in the UK, 81% of ophthalmologists doing provocative testing for PACS patients used pupillary dilation and only 15% used the dark room provocative test.^[32]^ Nevertheless, we explained the risks of the test to all patients. Finally, there was a difference in the sex ratio between both groups, but in the multiple regression analysis, the only determinant factor affecting the IOP changes of the provocative tests were the tests. Another limitation could be the lack of long-term follow-up of the patients and the correlation of the final glaucoma status with the initial test results; however, conducting such a study would be difficult to execute.

In conclusion, both pharmacologic mydriasis and WDT resulted in IOP elevation before LPI, but after LPI the IOP elevation was greater in the WDT group, which could be explained by the resolution of the pupillary block and consequently the lower IOP elevation in the pharmacologic mydriasis group. The mechanism of IOP elevation of WDT does not seem to be related to the biometric changes observed in PACS patients. Pharmacologic mydriasis and WDT may involve different mechanisms for IOP elevation, which may have the potential to advance our understanding about the mechanism of angle-closure glaucoma.

##  Financial Support and Sponsorship

Nil.

##  Conflicts of Interest

There are no conflicts of interest.
